# Longitudinal relationships among career adaptability, resilience, and career commitment in chinese nursing undergraduates: testing differences in career interest between cross-lagged models

**DOI:** 10.1186/s12912-023-01224-w

**Published:** 2023-03-24

**Authors:** Jingjing Zhang, Chengjia Zhao, Feiyue Li, Xiaoyi Wang, Huihui Xu, Miaomiao Zhou, Yiru Huang, Yeqin Yang, Guoliang Yu, Guohua Zhang

**Affiliations:** 1 School of Nursing, Wenzhou Medical University, 325035 Wenzhou, China; 2 School of Education, Renmin University of China, 100872 Beijing, China; 3 Department of Psychology, School of Mental Health, Wenzhou Medical University, 325035 Wenzhou, China; 4 Department of Thyroid Surgery, The First Affiliated Hospital of Wenzhou Medical University, 325015 Wenzhou, China; 5 Operating room of the Third Affiliated Hospital of Wenzhou Medical University, 325200 Wenzhou, China; 6 Key Research Center of Philosophy and Social Sciences of Zhejiang Province (Institute of Medical Humanities), Wenzhou Medical University, Wenzhou, 325035 China; 7 The Affiliated Kangning Hospital, Wenzhou Medical University, 325035 Wenzhou, China

**Keywords:** Career adaptability, Resilience, Career commitment, Career interest, Chinese nursing undergraduates, Cross-lagged model

## Abstract

**Purpose:**

Various physiological and psychological negative situations experienced by nurses as a result of COVID-19 pandemic have been shown to increase their perception of organizational difficulty and decrease their career commitment, thereby accelerating the turnover rate of nurses. Resilience and career adaptability have important influences on career commitment, so there is a need to evaluate the relationships between them and the underlying mechanisms.

**Patients and methods:**

Using a cross-lagged design, the Career Adaptability Scale, the Chinese version of career commitment, and Davidson’s Resilience Scale as research methods, we studied 692 nursing students for two consecutive years to evaluate the relationship among career adaptability, resilience, and career commitment.

**Results:**

Career adaptability at T1 substantially and positively predicts the career commitment at T2. Career adaptability and resilience are mutually predictive. No interaction is found between resilience and career commitment over time. There is a substantial difference in the cross-lagged relationship among career adaptability, resilience, and career commitment for low- and high-career interest.

**Conclusion:**

Our results show the importance of developing career commitment early on. Developing career adaptability, enhancing resilience, and increasing career interest in nursing students might help to increase career commitment.

## Introduction

Current economic developments have constrained the work patterns and reduced the resources, causing the health professionals to experience persistent symptoms of tiredness and discontent; it has increased the number of conflicts, stress, professional wear, and discontent [1–3]. Because of the close interaction between nurses and patients, nurses are regarded as one of the professionals with the highest risk of suffering from the so-called burnout syndrome [4, 5]. On an individual level, the career commitment that has been identified as a relevant factor in the studies addressing these issues [6]. COVID-19 pandemic has subjected health systems to unprecedented pressure, challenging their workforce, especially nurses [7].

Career commitment is defined as the harmony between an individual’s beliefs and professional goals; a higher harmony leads to a greater personal effort [8, 9]. Numerous factors influence career commitment, including socio-demographic traits (e.g., age, gender, level of education, voluntary choice of one’s major; [10, 11]), resilience [12], career adaptability [13, 14]. However, most studies focused on medical students, whereas only a few studies focused on nursing students. The majority of future nursing teams will be comprised of nursing students; thus, it is essential to evaluate the elements that influence their career commitment [15]. Although some empirical studies have found that resilience, career adaptability are positively related to career commitment in recent years, these studies have used a crosssectional approach [12, 13]. To fill these gaps, the current study used a crosslagged panel model to explore the longitudinal and bidirectional relationships among career adaptability, resilience, and career commitment. Moreover, we further examined the moderating effect of career interest among these relationships.

### Career adaptability and career commitment

Prior studies revealed that a high level of career adaptability in college students might increase learning, overcome obstacles, and facilitate to achieve future goals, and it was considered to be positively associated with academic engagement [16]. Career adaptability is considered as the readiness to cope with changes related to vocational development task, occupational transitions, and personal traumas [17]. Studies show that career adaptability is positively associated with career success, employee well-being [18], and career commitment [14]. According to the career construction theory [19], career adaptability is an adaptability resource that might impact the adaptation responses and outcomes [20]. Career commitment has been proven to be an adaptable response [21]. Based on this hypothesis, several empirical studies show that career adaptability contributes to the growth of levels of career commitment. For instance, HR Woo [22] found that career adaptability can enhance career commitment. In turn, employees with a high career commitment will be more motivated to do their job, be more adaptable to potential problems, and help their organizations succeed [23]. A previous study has identified career commitment as an important catalyst for career adaptability [13]. High levels of career commitment enable individuals to cope with future changes, and as a result these individuals are more likely to be successful in their organizations’ potential careers [24]. People with a very low career commitment find it difficult to make enough effort in their work or set high goals [25]. A longitudinal study showed that career adaptability and career commitment predict each other within three years [26]. Therefore, this study hypothesizes that nursing students’ career adaptability would have a reciprocal and positive relationship with career commitment over time (H1).

### Career adaptability and resilience

Career adaptability correlates favorably and strongly with resilience [27–29]. Resilience is defined as positive adaptability or the capacity to retain or regain mental health despite hardship [30]. J Rossier [31] defined resilience as the ability to handle stressful situations, as an adaptive response affected by adaptability. Positive psychological characteristics associated with optimism, hope, and resilience are also associated with career adaptability [32]. Studies showed that career adaptability is the foundation of resilience [29]. Moreover, studies showed that resilience mediates the relationship between career adaptability and life satisfaction [27, 28]. In a recent study, J Bimrose and L Hearne [33] focused on the relationship between resilience and career adaptability. Similarly, CR Wanberg and JT Banas [34] found that resilient employees were also better able to adapt to a professional change. The findings regarding resilience indicate that it is one of the strongest predictors of career adaptability [32, 35]. Therefore, this study hypothesizes that nursing students’ career adaptability would have a reciprocal and positive relationship with resilience over time (H2).

### Resilience and career commitment

Studies have shown that resilience is a substantial predictor of a variety of negative professional attitudes, including low-career identity, burnout, and low career commitment [36, 37]. According to the job demand-resource (JD-R) paradigm [38], resilience, a substantial psychological resource, might play an important role in initiating the motivational process and enhancing the career commitment [39]. When confronted with change, people with a high level of resilience typically have a more positive attitude, and they are more hopeful about the company’s future [40]. Their affective organizational commitment could be strengthened by their psychological desire to remain with the organization [41]. In addition, numerous studies have shown that resilience can accurately predict professional dedication [42, 43]. Meanwhile, scholars have argued that commitment is a crucial element of the resilience of entrepreneurs [33]. Some studies showed that people with a higher degree of career commitment are the most resilient [44, 45]. Therefore, this study hypothesizes that nursing students’ resilience would have a reciprocal and positive relationship with career commitment over time (H3).

### Differences in career interest

Career interest is defined as relatively persistent individual changes that influence behavior through preferences for particular work activities and work environments [46]. Career interest is a major indicator of diverse life outcomes, educational decisions, and career choices [47]. Furthermore, study showed that increasing career interest through career counseling can successfully promote career commitment, i.e., substantial differences are found in career commitment between individuals with high- and low-career interest [48, 49].

According to London’s model of career motivation, one of the personal traits, interest, has a direct impact on the surrounding environment. The more stable a person’s career interest are and the more they are integrated into their self-concept, the greater the direct influence of career interest on their career choices and behavior [50]. In other words, the greater the career interest, the greater the effect of career adaptability on career commitment. According to social cognitive theory, environmental variables can influence an individual’s behavior through internal factors [51]. As a personal trait of nursing students, career interest can facilitate the growth of skills, i.e., resilience as a skill [30]. In other words, career interest helps in the development of resilience. There is also empirical evidence that resilient students heavily rely on their enthusiasm for academic subjects to succeed [52]. Therefore, it is necessary to evaluate the differences in the relationship among career adaptability, resilience, and career commitment in terms of career interest. Therefore, this study hypothesizes substantial differences in career interest in the cross-lagged relationship among career adaptability, resilience, and career commitment (H4).

### This study

The relationships among career adaptability, resilience, and career commitment could be complex and have not been well studied. This two-wave longitudinal study aimed to elucidate these relationships in Chinese nursing undergraduates. We hypothesized that:

- H1: Career adaptability would have a reciprocal and positive relationship with career commitment over time.
- H2: Career adaptability would have a reciprocal and positive relationship with resilience over time.
- H3: Resilience would have a reciprocal and positive relationship with career commitment over time.
- H4: Substantial career interest differences in the cross-lagged relationship among career adaptability, resilience, and career commitment.

## Method

### Participants

This longitudinal study adopted a convenience sample recruited from 1 university in China. The inclusion criteria of this study included: (1) willing to participate in the baseline and follow-up studies; and (2) being about to enter an internship. The exclusion criteria included: (1) non-Chinese speaker; and (2) having cognitive impairment to understand the survey questions. This study used a follow-up study design with the first administration in June 2021 (T1, 823) and then a follow-up measurement one year apart in June 2022 (T2, 795). A total of 692 students completed all two waves of the survey. The two research coordinators indicated to the participants that participation is voluntary, and that there would be no negative consequence for declining to participate. Additionally, they ensured data confidentiality and clarified that only researchers would have access to the data. The participants received no monetary compensation.

### Measurements

#### Demographic measurements

Career interest was measured using the level of enjoyment of nursing profession.

#### Career adaptability

College students’ career adaptability was measured using the 21-item career adaptability Scale [53]. A sample item is “When faced with job options, I can make the right decision.” The questionnaire utilized Likert’s 5-point scoring, ranging from ‘very consistent’ to ‘very inconsistent’ with 5–1 point, respectively. To minimize confusion, ‘somewhat consistent’ was changed to ‘generic’ in the original questionnaire. The higher the score, the higher the level of career adaptability. The scale is reliable and valid, and it can be used to evaluate career adaptation in the Chinese cultural setting [10, 25]. The Cronbach’s alpha of the scale was 0.95 at T1 and 0.94 at T2.

#### Resilience

Resilience was measured using the Chinese version of Connor and Davidson’s resilience Scale [54]. The scale consists of 25 items; the three-factor structure of resilience can be derived from them: tenacity, strength, and optimism. Respondents evaluated the correspondence between the descriptions on the questionnaire and their circumstances and then rated the statements on a 5-point Likert scale ranging from 1 (totally false) to 5 (absolutely true). The higher the score, the greater the resilience. With a Cronbach alpha of 0.97 at T1 and 0.98 at T2.

#### Career commitment

The 27-item Undergraduate career commitment Scale [55] was used to measure the four-dimensional career commitment of college students. In the 5-point Likert scoring method, the scores range from 1 (totally disagree) to 5 (absolutely agree), where the higher the score, the greater the commitment to the related characteristic. The reliability and validity of this scale’s applicability to Chinese college students are excellent. The Cronbach’s alpha of the scale is 0.96 at T1 and 0.97 at T2.

### Data analysis

SPSS 23.0 software was used for data preprocessing, descriptive statistics, and correlation analysis. MPlus 8.3 software was used to construct structural equation models for the latent variables in the Cross-lagged analysis, and the model was estimated using the robust weighted least square estimator (WLSMV) available [56], and holographic great likelihood estimation was used for the missing data [57]. Since age is a well-documented factor in career commitment [58, 59], it was adjusted for in the model. The fit of all the models was evaluated using various indices as operationalized in Mplus in combination with the WLSMV estimator [60]: the WLSMV Chi-square statistic (χ^2^), the Comparative Fit Index (CFI), the Tucker Lewis Index (TLI), the Root-Mean-Square Error of Approximation (RMSEA) and its 90% confidence interval. These fit indices are interpreted as in ML/MLR estimation with the value of CFI and TLI greater than 0.90, and RMSEA smaller than 0.08 indicates acceptable model fit.

## Results

### Preliminary analyses

A total of 307 participants had high-career interest, and a total of 385 participants had low-career interest.

We compared the characteristics of those who were followed up (*n* = 692) versus those who were missing in the first and second follow-up surveys (*n* = 103). The two groups did not differ in socio-demographic characteristics or the levels of the independent and dependent variables (*p* > .05).

A common method for bias testing was performed. According to Harman’s single-factor test, 50 principal components were extracted without rotation, and the explanatory rate of the total variance variation of the first was 38.5%, lower than the critical value of 40.0% [61]. Thus, the data had no severe common bias.

As shown in Table 1, all the variables at each time point had significant and positive correlations (see Table 1).

**Table 1:** Descriptive statistics and correlations among variables of interest

		M	SD	1	2	3	4	6	7	8	9	11	12	13	15	16	17	19	20	21	22	24	25	26	27
CA1	CA11	17.82	2.84	1																					
	CA12	18.26	2.81	0.89^***^	1																				
	CA13	17.58	2.60	0.88^***^	0.88^***^	1																			
	CA14	17.86	2.64	0.87^***^	0.89^***^	0.87^***^	1																		
CA2	CA21	18.48	3.37	0.38^***^	0.41^***^	0.39^***^	0.39^***^	1																	
	CA22	18.49	2.96	0.40^***^	0.45^***^	0.42^***^	0.42^***^	0.87^***^	1																
	CA23	18.05	2.98	0.40^***^	0.45^***^	0.43^***^	0.43^***^	0.89^***^	0.90^***^	1															
	CA24	18.09	2.86	0.39^***^	0.44^***^	0.41^***^	0.43^***^	0.88^***^	0.89^***^	0.90^***^	1														
RE1	RE 11	31.38	4.88	0.75^***^	0.68^***^	0.73^***^	0.70^***^	0.37^***^	0.38^***^	0.40^***^	0.37^***^	1													
	RE 12	28.21	4.79	0.77^***^	0.73^***^	0.77^***^	0.74^***^	0.38^***^	0.39^***^	0.40^***^	0.39^***^	0.91^***^	1												
	RE 13	25.43	4.19	0.78^***^	0.74^***^	0.76^***^	0.75^***^	0.38^***^	0.41^***^	0.42^***^	0.41^***^	0.91^***^	0.92^***^	1											
RE2	RE 21	32.45	5.60	0.33^***^	0.35^***^	0.33^***^	0.34^***^	0.81^***^	0.71^***^	0.74^***^	0.74^***^	0.38^***^	0.36^***^	0.36^***^	1										
	RE 22	29.28	5.43	0.36^***^	0.39^***^	0.38^***^	0.38^***^	0.83^***^	0.73^***^	0.77^***^	0.77^***^	0.40^***^	0.42^***^	0.41^***^	0.94^***^	1									
	RE 23	26.11	4.53	0.37^***^	0.41^***^	0.38^***^	0.39^***^	0.84^***^	0.77^***^	0.79^***^	0.79^***^	0.40^***^	0.42^***^	0.42^***^	0.94^***^	0.95^***^	1								
CC1	CC11	24.90	3.85	0.69^***^	0.70^***^	0.70^***^	0.72^***^	0.32^***^	0.36^***^	0.37^***^	0.36^***^	0.60^***^	0.65^***^	0.67^***^	0.28^***^	0.32^***^	0.32^***^	1							
	CC12	21.36	3.06	0.63^***^	0.66^***^	0.66^***^	0.70^***^	0.29^***^	0.35^***^	0.33^***^	0.33^***^	0.56^***^	0.59^***^	0.63^***^	0.24^***^	0.29^***^	0.30^***^	0.88^***^	1						
	CC13	24.48	3.44	0.64^***^	0.65^***^	0.65^***^	0.69^***^	0.32^***^	0.36^***^	0.34^***^	0.35^***^	0.60^***^	0.64^***^	0.66^***^	0.28^***^	0.31^***^	0.32^***^	0.89^***^	0.87^***^	1					
	CC14	20.81	3.54	0.65^**^	0.62^***^	0.64^***^	0.64^***^	0.31^***^	0.35^***^	0.34^***^	0.34^***^	0.62^***^	0.64^***^	0.63^***^	0.28^***^	0.30^***^	0.30^***^	0.88^***^	0.82^***^	0.86^***^	1				
CC2	CC21	25.03	4.31	0.32^***^	0.38^***^	0.34^***^	0.37^***^	0.78^***^	0.74^***^	0.78^***^	0.77^***^	0.30^***^	0.32^***^	0.32^***^	0.74^***^	0.76^***^	0.78^***^	0.40^***^	0.37^***^	0.39^***^	0.38^***^	1			
	CC22	21.40	3.43	0.33^***^	0.39^***^	0.35^***^	0.37^***^	0.74^***^	0.71^***^	0.75^***^	0.74^***^	0.30^***^	0.32^***^	0.33^***^	0.70^***^	0.73^***^	0.75^***^	0.41^***^	0.41^***^	0.40^***^	0.39^***^	0.91^***^	1		
	CC23	24.69	3.74	0.35^***^	0.40^***^	0.36^***^	0.38^***^	0.79^***^	0.73^***^	0.77^***^	0.76^***^	0.33^***^	0.35^***^	0.35^***^	0.76^***^	0.77^***^	0.79^***^	0.41^***^	0.39^***^	0.42^***^	0.40^***^	0.91^***^	0.89^***^	1	
	CC24	21.35	4.19	0.30^***^	0.32^***^	0.30^***^	0.32^***^	0.77^***^	0.67^***^	0.73^***^	0.72^***^	0.26^***^	0.28^***^	0.27^***^	0.75^***^	0.76^***^	0.75^***^	0.37^***^	0.34^***^	0.37^***^	0.40^***^	0.92^***^	0.88^***^	0.90^***^	1
	V	0.44	− 0.06	− 0.08^*^	− 0.06	− 0.06	− 0.05	− 0.04	− 0.04	− 0.05	− 0.09^*^	− 0.09^*^	− 0.10^*^	− 0.05	− 0.05	− 0.04	− 0.07	− 0.08^*^	− 0.07	− 0.08	− 0.07	− 0.05	− 0.05	− 0.05	− 0.04

*CA11*  Career confidence at T1, *CA12 * career curiosity at T1, *CA13 * career focus at T1, *CA14 *career control at T1, *CA1 *career adaptability at T1, *RE1 *resilience at T1, *RE 11 *Toughness at T1, *RE 12 *strength at T1, *RE 13 *optimism at T1, *RE 1 *RE at T1; *CC11 *Emotional commitment at T1, *CC12 *continuing commitment at T1, *CC13 *aspirational commitment at T1, *CC14 *normative commitment at T1, *CC1 *career commitment at T1. *CA21 *Career confidence at T2, *CA22 *career curiosity at T2, *CA23 *career focus at T2, *CA24 *career control at T2, *CA2 *career adaptability at T2; *RE2 *resilience at T2; *RE 21*  Toughness at T2, *RE 22 *strength at T2, *RE 23 *optimism at T2, *RE 2 *RE at T2; *CC21 *Emotional commitment at T2, *CC22 *continuing commitment at T2, *CC23 *aspirational commitment at T2, *CC24 * normative commitment at T2, *CC2 *career commitment at T2. *V *voluntary choice of the nursing major

^***^, *p* < .001; , *p* < .01; , *p* < .05^**^^*^

### Model testing

This study used the latent variable structural equation model of Mplus 8.3 to evaluate the cross-lagged relationship between career adaptability, resilience, and career commitment. It was first essential to check the measurement invariance of career adaptability, resilience, and career commitment across the two measurements. The model fits are shown in Table 2. GW Cheung and RB Rensvold [62] recommended to use the indicator ΔCFI in model comparisons. This indicator is independent of model parameters and sample size. When ΔCFI is less than or equal to 0.01 (0.003, 0.004), then the assumption of measurement invariance is accepted, thus allowing this measurement model to be further analyzed across lags.

**Table 2:** The measurement invariance of the scale over time

Model	*χ*^*2*^	*df*	*p*	CFI	TLI	RMSEA (90%CI)	SRMR	ΔCFI	ΔTLI
Step1	976.26	205	< 0.001	0.968	0.961	0.071[0.069, 0.079]	0.042		
Step2	1060.36	213	< 0.001	0.965	0.959	0.076[0.071, 0.080]	0.060	0.003	0.005
Step3	1161.22	224	< 0.001	0.961	0.956	0.078[0.073, 0.082]	0.060	0.004	0.002

*Step 1 *Configural Invariance, *Step 2 *Weak Factorial Invariance, *Step 3 *Strong Factorial Invariance. *CFI *Comparative Fit Index, *TLI *Tucker, and Lewis Index, *RMSEA *Root Mean Squared Error of Approximation

### Cross-lagged analysis of career adaptability, resilience, and career commitment

**Fig. 1:**
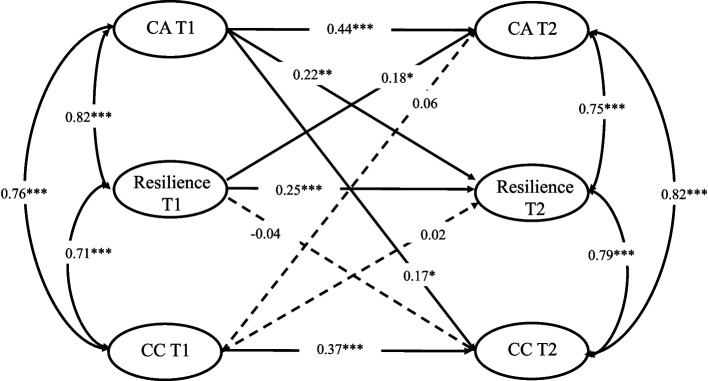
Cross-lagged path diagram Note. All the reported parameters are standardized. CA: career adaptability; CC: career commitment. * *p* < .05, ** *p* < .01, *** *p* < .001

Figure 1 shows the model estimation results. The cross-lagged model fitted the data well (*χ*^2^ = 1028.265, *df* = 205, *p <* .001; CFI = 0.966, TLI = 0.962, RMSEA = 0.076, SRMR = 0.056; Fig. 1). After controlling for age, the results of autoregressive path analysis of the same variables at different time points show that career adaptability at T1 significantly predicted career adaptability at T2 (*β* = 0.44, *p <* .001); resilience at T1 significantly predicted resilience at T2 (*β* = 0.25, *p <* .001); career commitment at T1 significantly predicted the career commitment at T2 (*β* = 0.37, *p <* .001). This indicates that the nursing students’ career adaptability, resilience, and career commitment are stable across time. More importantly, the results of cross-lagged path analysis show that career adaptability at T1 significantly predicted both resilience (*β* = 0.22, *p <* .01) and career commitment (*β* = 0.17, *p <* .05) at T2. Resilience at T1 significantly predicted career adaptability (*β* = 0.18, *p <* .05) but not career commitment (*β* = − 0.04, *p >* .05) at T2. Career commitment at T1 insignificantly predicted career adaptability (*β* = 0.06, *p >* .05) and resilience (*β* = 0.02, *p >* .05) at T2. Overall, career adaptability was a found to be a predictor of resilience and career commitment for nursing students, and resilience was also found to be a predictor of career adaptability.

### Comparison between low- and high-career interest in the cross-lagged model

**Fig. 2:**
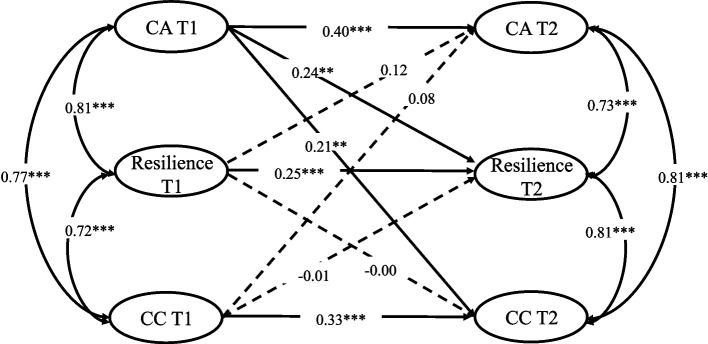
Cross-lagged path diagram (high-career interest group)

**Fig. 3:**
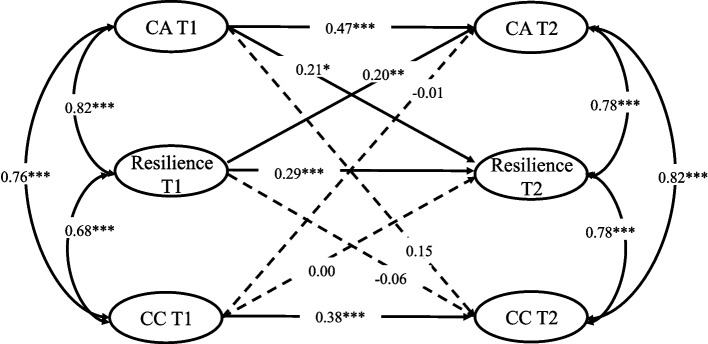
Cross-lagged path diagram (low-career interest group)

A multigroup comparative structural equation model was used to test the differences in the cross-lagged relationship between career adaptability, resilience, and career commitment in nursing students with low- and high-career interest. The results show (see Table 3) a significant difference between the chi-square test results of the control model and free estimation model, Δ*χ*^*2*^(8) = 67.71, *p* < .001. As a result, the model results for the high-career interest group showed significant differences from those for the low-career interest group. The results of multigroup comparison further indicate that the high-career interest group significantly differed from the low-career interest group on the pathway of career adaptability predicting career commitment: Wald Test of Parameter Constraints (*t* = 4.82, *p* < .05). Career adaptability in the high-career interest group at T1 significantly and positively predicted career commitment at T2 (b = 0.21, *p* < .01), whereas career adaptability in the low-career interest group at T1 did not significantly predict career commitment at T2 (b = 0.15, *p* > .05). No significant difference was observed in other pathway comparisons. The path coefficients of free estimation model for the high- and low-career interest groups are shown in Figs. 2 and 3.

**Table 3:** Model comparison between the low and high career interest groups

	Model fit								
	*χ*^*2*^	*df*	*p*	CFI	TLI	RMSEA	SRMR	ΔCFI	ΔTLI
Limited	1209.37	424	< 0.001	0.968	0.965	0.073	0.065		
Free estimation model	1277.08	432	< 0.001	0.965	0.963	0.075	0.078	0.003	0.002

## Discussion

In this study, we used a cross-lagged design to evaluate the relationship between career adaptability, resilience, and career commitment and the role of career interest. The results show an interaction between career adaptability, resilience, and career commitment in nursing students, and that this relationship differs in terms of low- and high-career interest groups.

### Longitudinal relationship between career adaptability and career commitment

In this study, we found that career adaptability at T1 significantly predicted career commitment at T2, but career commitment at T1 did not significantly predict career adaptability at T2. Thus, H1 was partially supported. Consistent with earlier studies [14, 63] demonstrating a direct relationship between career adaptability and career commitment. This outcome is also consistent with the career construction theory [19]. By increasing their career adaptability, nursing students can increase their professional knowledge and career confidence. After establishing expectations and objectives for themselves and their work position, students’ career commitment steadily increases [64]. Career commitment at T1 did not significantly predict career adaptability at T2, inconsistent with the results [13, 26], although the study showed that a higher career commitment appears to have considerable benefits for occupational adjustment [26]. Nursing students often face substantial occupational stress at the beginning of their clinical job, as evidenced by barriers to nurse–patient communication, lack of professional knowledge, and unfamiliarity with the clinical environment, resulting in anxiety and frustration, and decreased enthusiasm for the job [65, 66]. This may promote self-doubt about the current commitment [63]. Students with a high degree of career adaptability might constantly be enthusiastic about studying and applying the theory into practice. As a result, they are more motivated to work for the profession and have a higher aim and resolve to make contributions to the nursing profession. To effectively increase career commitment, it is important to make proper career preparations in advance through career planning education.

### Longitudinal relationship between career adaptability and resilience

Moreover, our study shows that career adaptability would have a reciprocal and positive relationship with resilience over time. Thus, H2 was supported. On one hand, previous cross-sectional studies have confirmed that they are closely related [28, 29]. This outcome is also consistent with the career construction theory [19]. This means that a set of personal resources that, together with one’s ability to actively overcome and cope with stressful situations, might contribute to resilience of nursing students after their job placement. These challenges are likely to involve not only exceptional or traumatic events [67], but also difficulties related to life routines, the nurse–patient relationship, the teacher–student relationship, and social and work uncertainty in the current social context [4, 5]. On the other hand, resilience at T1 significantly predicted career adaptability at T2, consistent with previous studies [32, 35]. Resilience is a positive personal characteristic that is widely referred to in both Chinese and Western cultures [32, 63, 68], and it is more likely to prepare students for future challenges [69, 70], especially the various situations faced during internships. Studies have also highlighted the role of resilience in career counseling to better cope with an unstable labor market [33]. Therefore, it is particularly important to increase the level of resilience of nursing students before and during their internship to improve career adaptability.

### Longitudinal relationship between resilience and career commitment

Our findings showed that resilience and career commitment are not mutually predictable. H3 was not supported. First, in contrast to earlier studies [37, 42, 43] and the job demand-resource (JD-R) model [38], In this study, a medium level of resilience at T1. This might have been influenced by the unfriendly environment, heavy workload, unsatisfactory pay, and limited opportunity for advancement, decreasing their resilience and affecting their career commitment [71]. To improve career commitment, a strong social support system is needed [45], Satya group counseling [72] is implemented, and a positive psychological intervention is provided [73]. Secondly, career commitment at T1 did not significantly predict resilience at T2. We interpreted this result in two ways. First, we did not use usual commitment (e.g., effective commitment) to evaluate their relationship with resilience. The role of career commitment to resilience might vary according to the classification [74]. For example, the findings suggest that affective commitment promotes resilience, whereas continued commitment does not promote resilience [64]. Secondly, there may be situational and/or cross-cultural differences. Malaysian managers were more collectivist but also more longitudinally individualistic than their Australian counterparts, with the two groups differing only in their resilience and career commitment levels [75]. This has not yet been evaluated in a Chinese sample.

### Differences in career interest

H4 was supported, specifically, career adaptability at T1 can significantly influence career commitment at T2 for high-career interest groups, whereas career adaptability at T1 cannot significantly influence career commitment at T2 for low-career interest groups. This suggests that the long-term predictive effect of career adaptability on career commitment might be influenced by other factors (e.g., high-career interest). This is probably because students with low-career interest do not know what they want; they are less able to self-regulate and less likely to derive satisfaction from their careers [76]. In particular, resilience at T1 did not predict career commitment at T2 when situations arise during the job placement [65, 66]. Interestingly, resilience at T1 significantly affects career adaptability at T2 for low-career interest groups, while resilience at T1 does not significantly affect career adaptability at T2 for high-career interest groups, with no significant difference in the pathway comparisons. Students with high levels of resilience rely on career interest to increase their career adaptability level [40]. The career performance of students with a limited resilience depends on their early career interest [52]. Therefore, the proportion of students who choose their careers should be increased to enhance their career performance [77]. In addition, nursing educators should integrate career education into students’ daily academic life prior to practice to increase their career adaptability level [63].

## Implications and limitation

Several limitations of this study must be recognized. First, we selected samples of college students only, which will result in a sampling bias; hence, the extrapolation of our results should be considered seriously. Future studies might extend the concept to a variety of populations. Second, there was only one grade level for the subjects, and it was not possible to compare age variability. Future studies should involve multigrade subjects. Third, in this study, we used a tracking survey research design. This design, to some extent, avoids the shortcomings of cross-sectional studies. However, we only measured two points in time, and the number of measurements could be increased to obtain stable trends in career adaptability, resilience, and career commitment. Therefore, future studies should further examine the stability of the relationship between the three multiple times. Fourth, in this study, career interest was illustrated by descriptive statistics. More standardized scales should be used for measurement in future studies. Finally, this study is not an intervention study, and future studies could incorporate an intervention research design to better evaluate whether developing career adaptability in nursing students is effective in increasing their level of career commitment.

Despite limitations, the current study has several important implications. From a theoretical perspective, the current study adds to the study of the temporal sequences among career adaptability, resilience, and career commitment. This will contribute to a better understanding of how and when career adaptability increases career commitment. From a practical perspective, the present study indicates that career adaptability may increase subsequent resilience and career commitment in undergraduate trainee nursing students. Furthermore, career interest made a significant difference in the relationship between career adaptability, resilience, and career commitment. Therefore, nursing educators should integrate career education into the daily academic life of students. For example, career education should be integrated into the career curriculum and the development of career adaptability should be incorporated into the content of lectures; nursing students should be given the necessary career training during their internships. Courses on resilience should also be offered to increase students’ cognitive-emotional regulation, behavioral training and mindfulness training; and social media platforms should be set up to provide intern nursing students with avenues for emotional support. These approaches allow for negative and unexpected events to be faced calmly and positive coping strategies to improve career commitment and become a quality reserve of clinical frontline nursing staff. Furthermore, given the difference between career interest in the relationship between career adaptability and career commitment, it suggests that educational administrators need to pay attention to career interest. Specifically, educators and practitioners should design effective ways to make learning a more meaningful and interesting experience, which has the potential to promote career engagement among nursing students who already show low interest. There is also a need to help nursing students understand the advantages of career adaptability and thus increase their career commitment.

## Conclusion

In summary, the following conclusions can be drawn: (1) Career adaptability and career commitment do not predict each other, and the career adaptability at T1 significantly and positively predicts the career commitment at T2. (2) Career adaptability and resilience are mutually predictive. (3) No interaction is found between resilience and career commitment. (4) There is a significant difference in the cross-lagged relationship among career adaptability, resilience, and career commitment for low- and high-career interest groups. Our results show the importance of developing career commitment early on. Developing career adaptability and enhancing resilience among nursing students might contribute to increased career commitment, for example, integrating career education into the professional curriculum and infusing the development of career adaptability into lectures [78], providing nursing students the necessary career training, resilience development sessions such as career group counseling, and cognitive–emotional regulation during practice [79, 80].

## Data Availability

The datasets in the study are available from the corresponding author on reasonable request.
